# Supplementary figures and images for: Exclusive dependence of IL-10Rα signalling on intestinal microbiota homeostasis and control of whipworm infection
Source: PLoS Pathog. 2019 Jan 14;15(1):e1007265. doi: 10.1371/journal.ppat.1007265 (PMC6347331; doi:10.1371/journal.ppat.1007265)

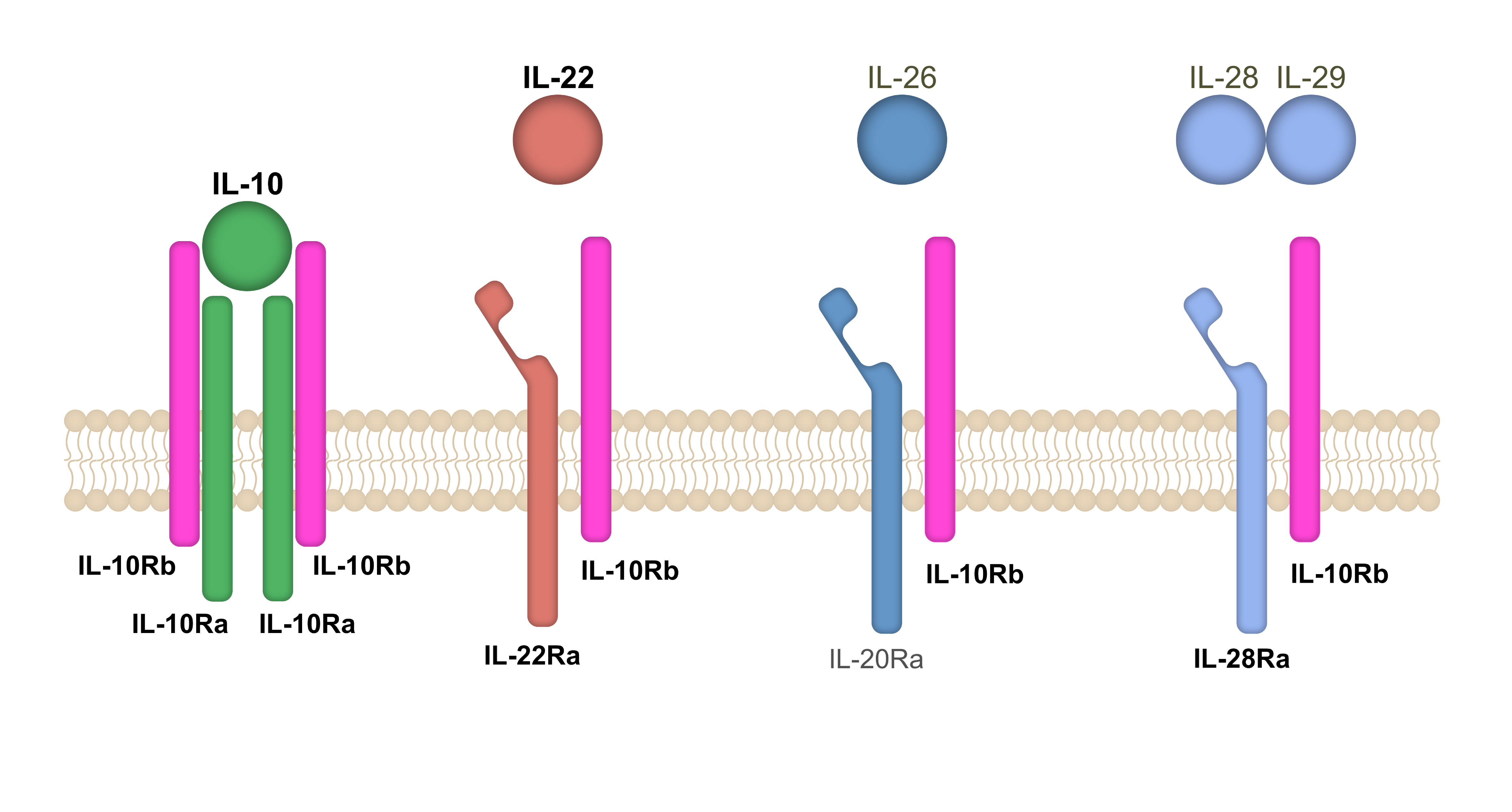

Supplement: S1 Fig — Schematic illustration showing IL-10 family of cytokines that share the IL-10Rβ chain as a subunit of their receptors. Highlighted in bold are the molecules for which mutant mice were available and used for experiments. (TIF) [file ppat.1007265.s001.tif]

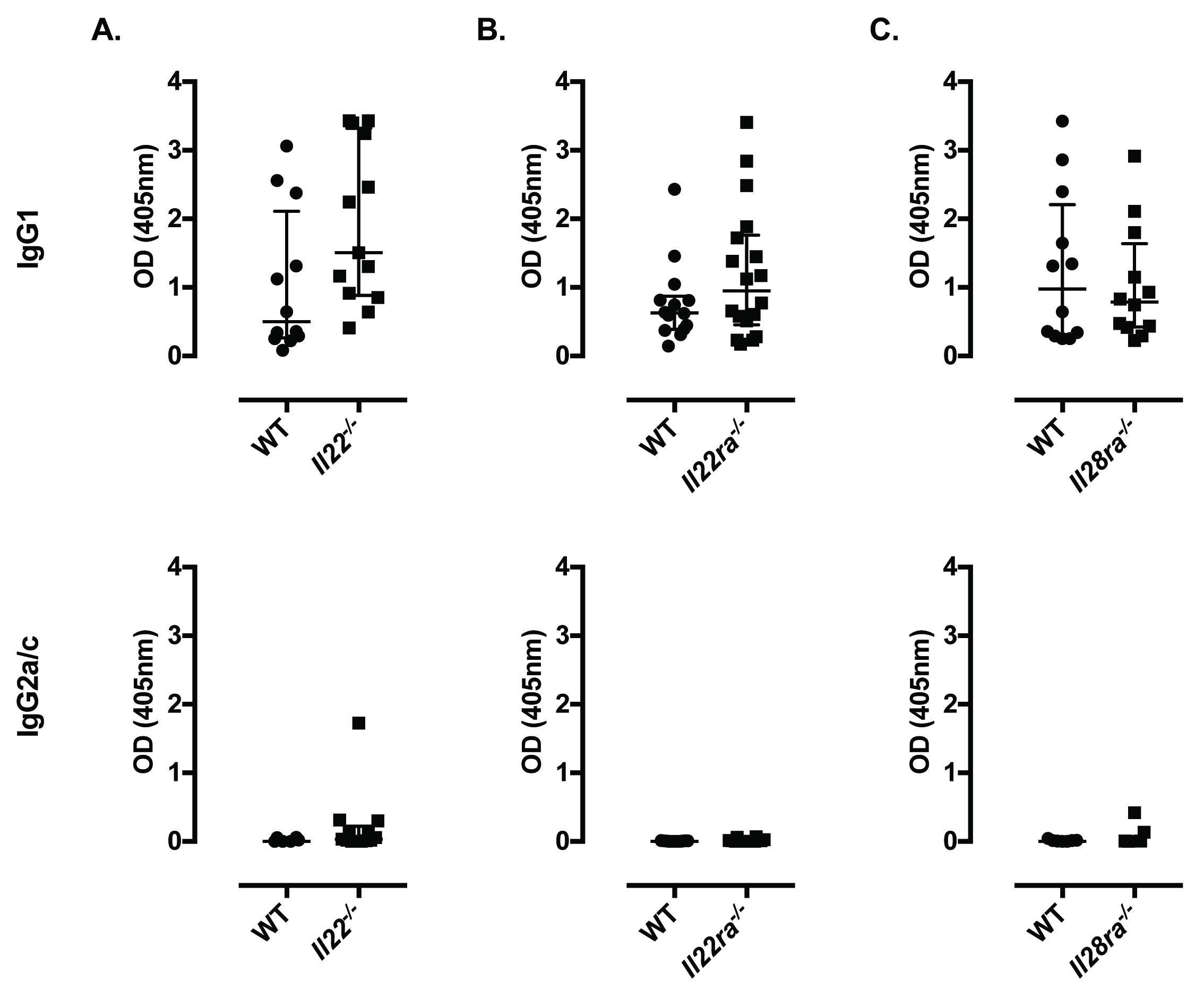

Supplement: S2 Fig — Antibody (IgG1 and IgG2a/c) titres of T. muris-infected, six to ten-wk-old female WT and (A) Il22-/-, (B) Il22ra-/- and (C) Il28ra-/- mice after 32 days of high dose infection (400 eggs). No differences in worm expulsion were observed at this time point. Data from two independent replicas. Median and interquartile range are shown. (A) WT n = 12, Il22-/- n = 13. (B) WT n = 14, Il22ra-/- n = 18. (C) WT n = 12, Il28ra-/- n = 12. (TIF) [file ppat.1007265.s002.tif]

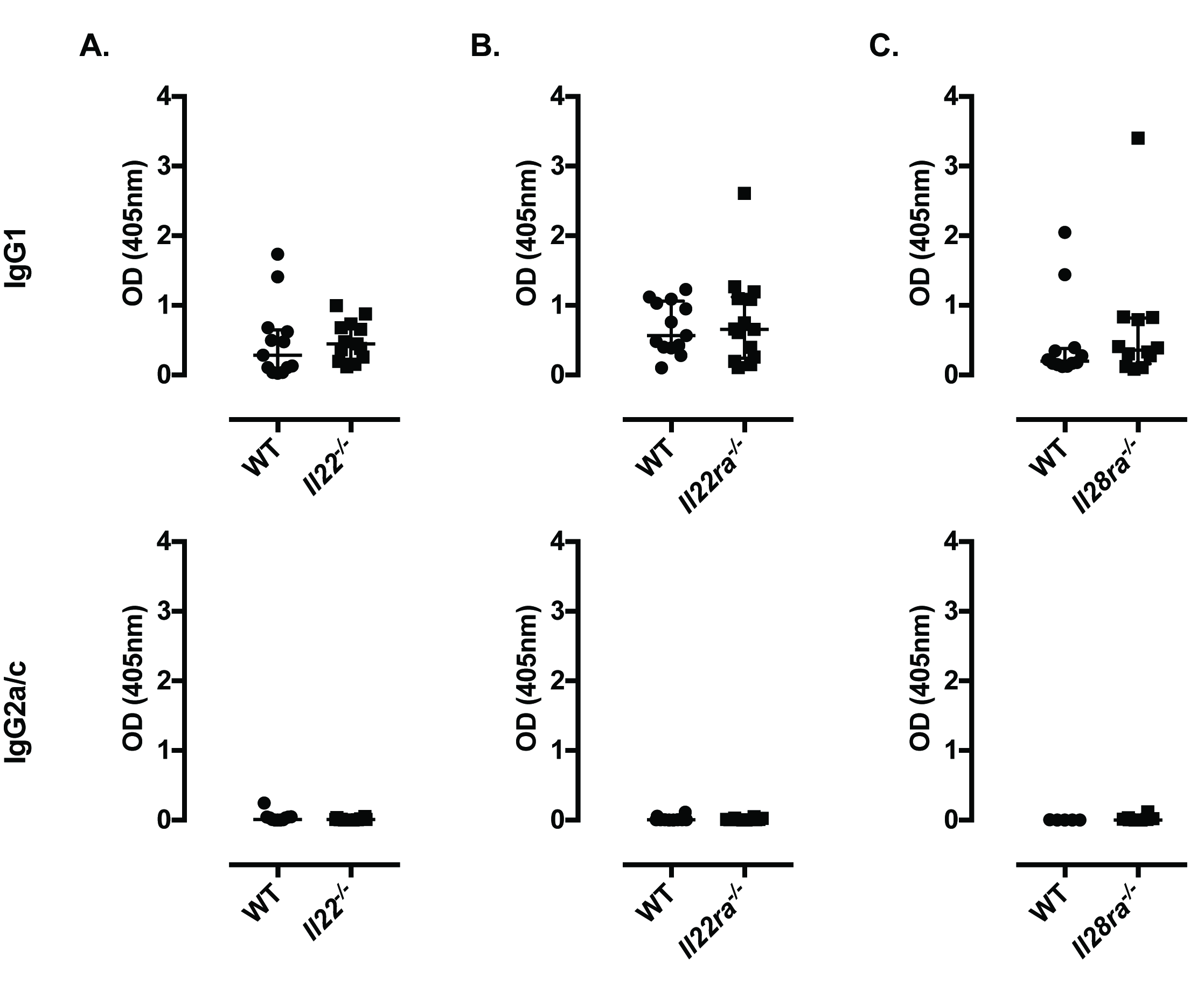

Supplement: S3 Fig — Antibody (IgG1 and IgG2a/c) titres of T. muris-infected, six to ten-wk-old female WT and (A) Il22-/-, (B) Il22ra-/- and (C) Il28ra-/- mice after 21 days of high dose infection (400 eggs). No differences in worm expulsion were observed at this time point. Data from two independent replicas. Median and interquartile range are shown. (A) WT n = 13, Il22-/- n = 13. (B) WT n = 13, Il22ra-/- n = 14. (C) WT n = 12, Il28ra-/- n = 12. (TIF) [file ppat.1007265.s003.tif]

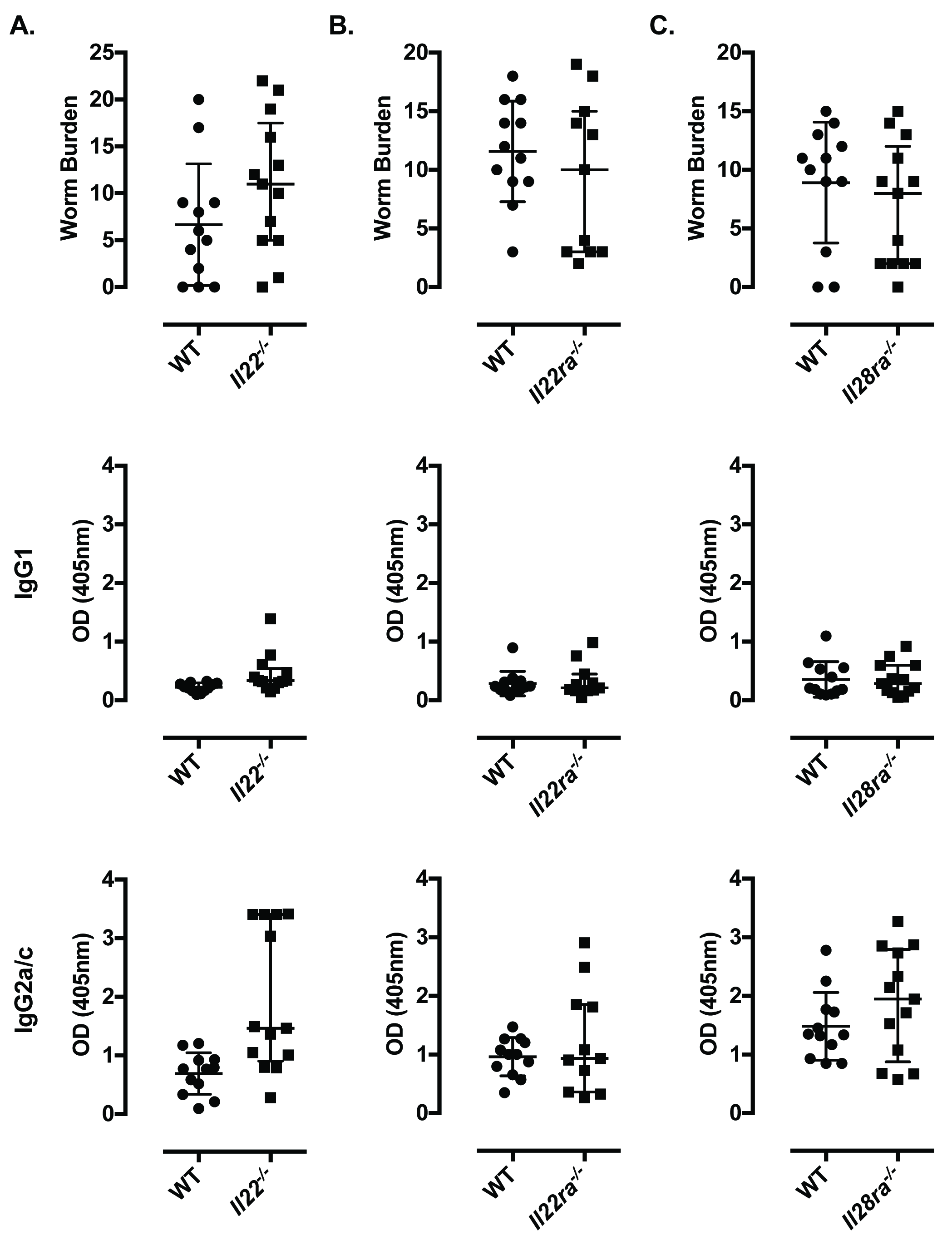

Supplement: S4 Fig — Worm burden and antibody (IgG1 and IgG2a/c) titres of T. muris-infected, six to ten-wk-old female WT and (A) Il22-/-, (B) Il22ra-/- and (C) Il28ra-/- mice after 35 days of low dose infection (20–25 eggs). Data from two independent replicas. Median and interquartile range are shown. (A) WT n = 12, Il22-/- n = 13. (B) WT n = 12, Il22ra-/- n = 11. (C) WT n = 12, Il28ra-/- n = 13. (TIF) [file ppat.1007265.s004.tif]

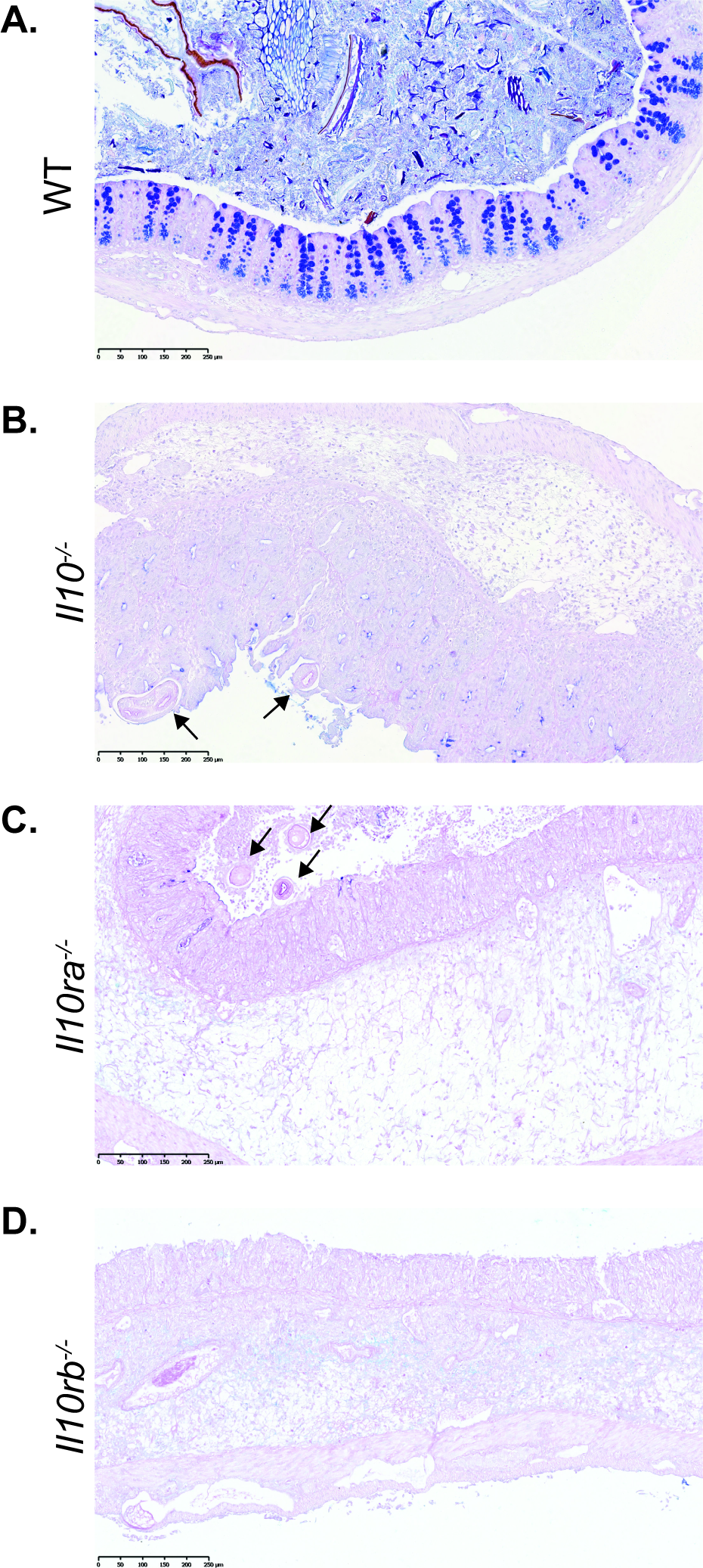

Supplement: S5 Fig — Representative images of PAS staining on caecum sections of T. muris-infected (high dose, 400 eggs) (A) WT, (B) Il10-/-, C) Il10ra-/- and (D) Il10rb-/- mice upon culling. Infected WT mice present goblet cell hyperplasia while infected IL-10 signalling-deficient mice show goblet cell loss. T. muris worms are infecting the mucosa (arrows) of IL-10 signalling-deficient mice. Scale bar, 250μm. Data from two independent replicas (n = 5–10 each). (TIF) [file ppat.1007265.s005.tif]

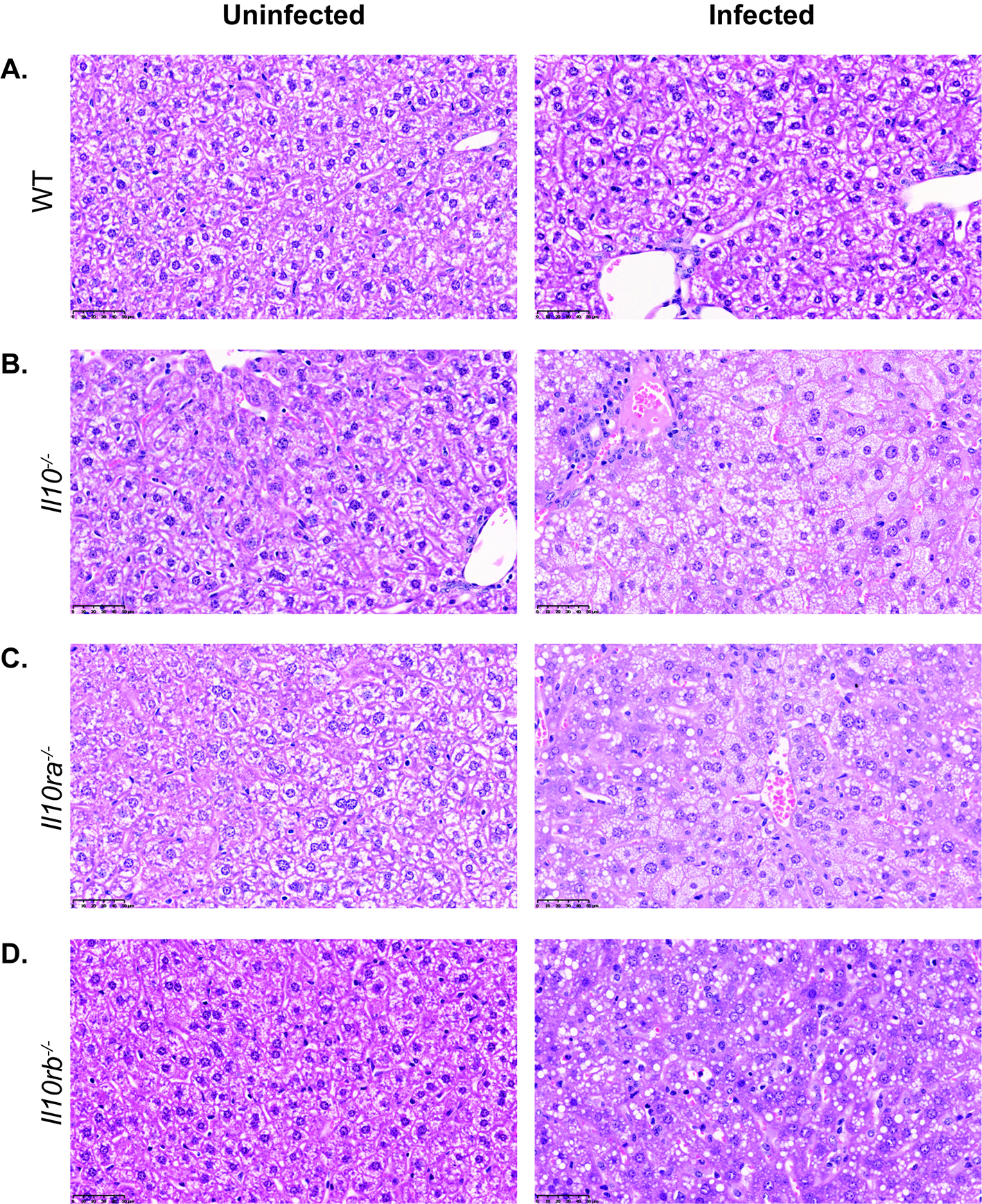

Supplement: S6 Fig — Liver histopathology of uninfected and T. muris-infected (high dose, 400 eggs) (A) WT, (B) Il10-/-, (C) Il10ra-/- and (D) Il10rb-/- mice upon culling. Sections stained with H&E. Uninfected WT and mutant mice show no lesions. Upon infection, some IL-10 signalling-deficient mice show inflammatory infiltrate characterized by foamy macrophages. Scale bar, 50μm. Data from two independent replicas (n = 5–18 each). (TIF) [file ppat.1007265.s006.tif]

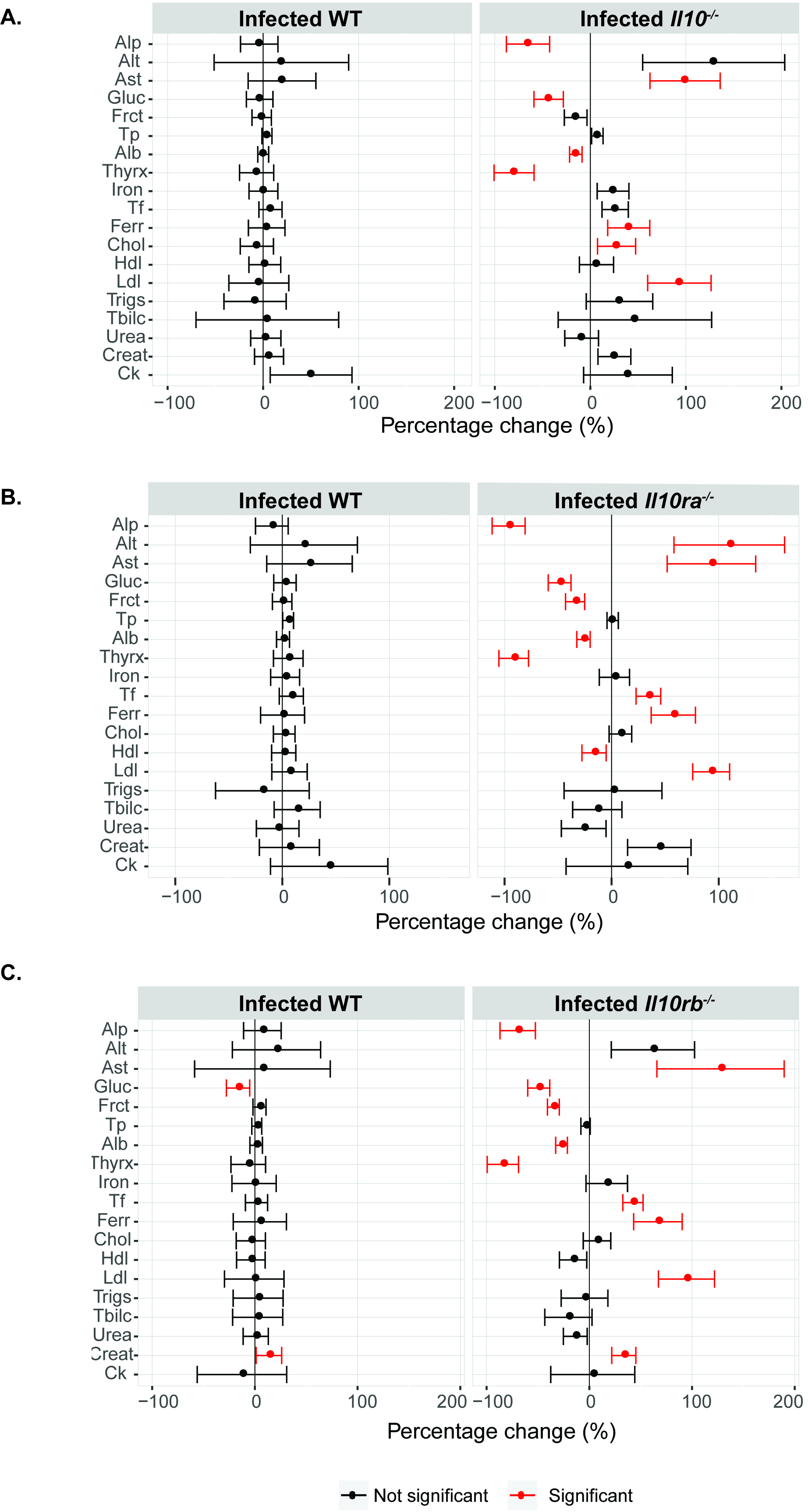

Supplement: S7 Fig — Percentage change of plasma chemistry parameters upon culling of T. muris-infected (high dose, 400 eggs), six-wk-old female and male littermate WT and (A) Il10-/-, (B) Il10ra-/-, (C) Il10rb-/- mice. The infection status effect on each genotype for plasma chemistry parameters associated with liver disease was estimated across independent experiments. The estimate has been visualised as a percentage normalised value (signal/average signal for that parameter) along with the 95% confidence interval to allow comparison across variables. Highlighted in red, are parameters where the genotype by infection is statistically significant in explaining variation after adjustment for multiple testing (5% FDR) and are significant in the final model estimate (p<0.05). (A) Data from three independent replicas. WT n = 24. Il10-/- n = 23. (B) Data from three independent replicas. WT n = 25. Il10ra-/- n = 22. (C) Data from two independent replicas. WT n = 16. Il10rb-/- n = 18. Alkaline phosphatase (Alp), aspartate aminotransferase (Ast), alanine aminotransferase (Alt), glucose (Gluc), fructosamine (Fruct), total protein (Tp), albumin (Alb), thyroxine (Thyrx), transferrin (Tf), ferritin (Ferr), cholesterol (Chol), high density lipoprotein (Hdl), low density lipoprotein (Ldl), triglycerides (Trigs), total bilirubin (Tblic), urea, creatinine (Creat) and creatinine kinase (CK). (TIF) [file ppat.1007265.s007.tif]

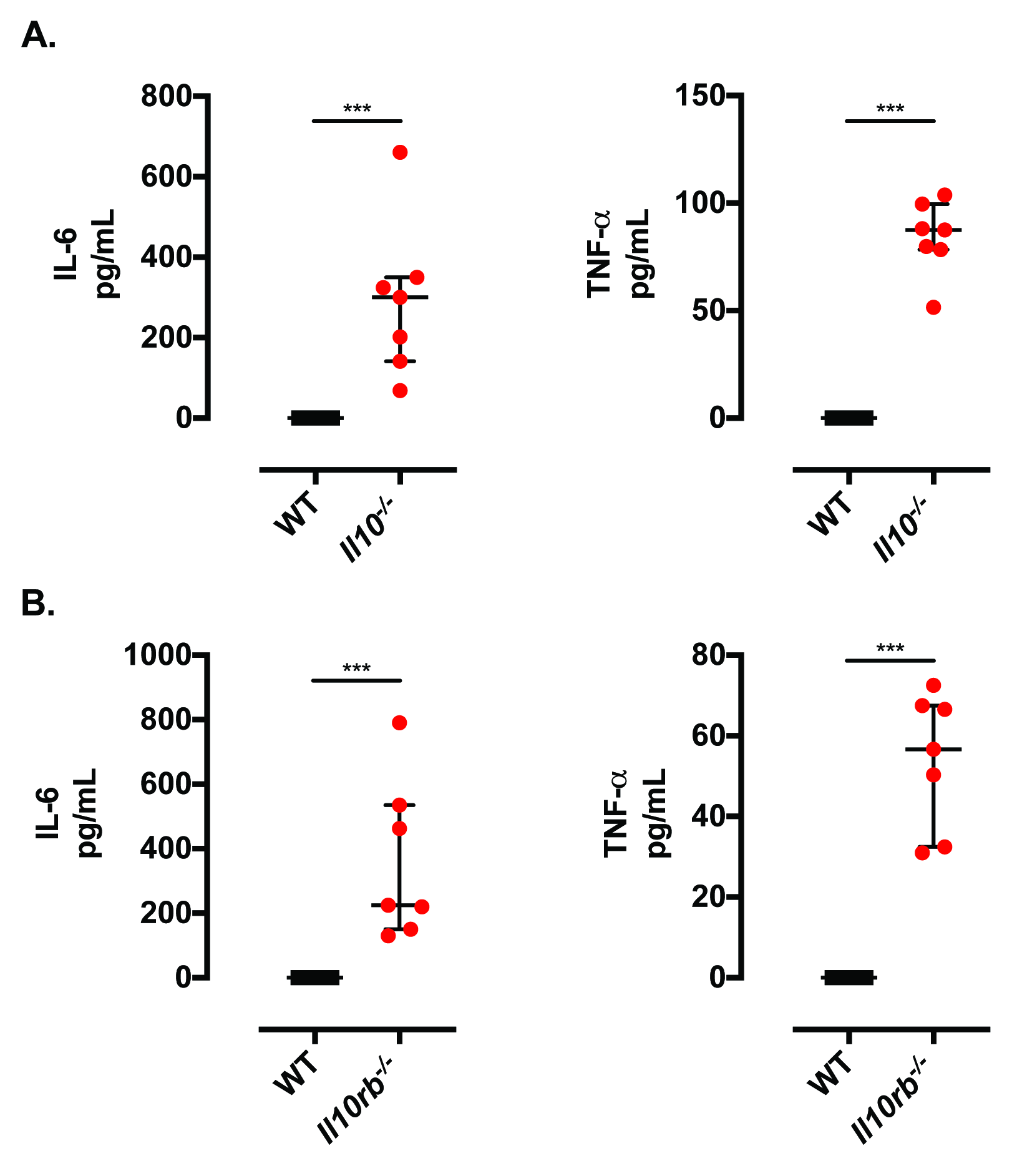

Supplement: S8 Fig — IL-6 and TNF-α concentrations in plasma of T. muris-infected (high dose, 400 eggs), six-wk-old female and male littermate WT and (A) Il10-/- and (B) Il10rb-/- mice. (A) Data from two independent replicas. WT n = 7. Il10-/- n = 7. Median and interquartile range are shown. Mann Whitney U Test, ***p<0.001. (B) Data from two independent replicas. WT n = 7. Il10rb-/- n = 7. Median and interquartile range are shown. Mann Whitney U Test, ***p<0.001. (TIF) [file ppat.1007265.s008.tif]

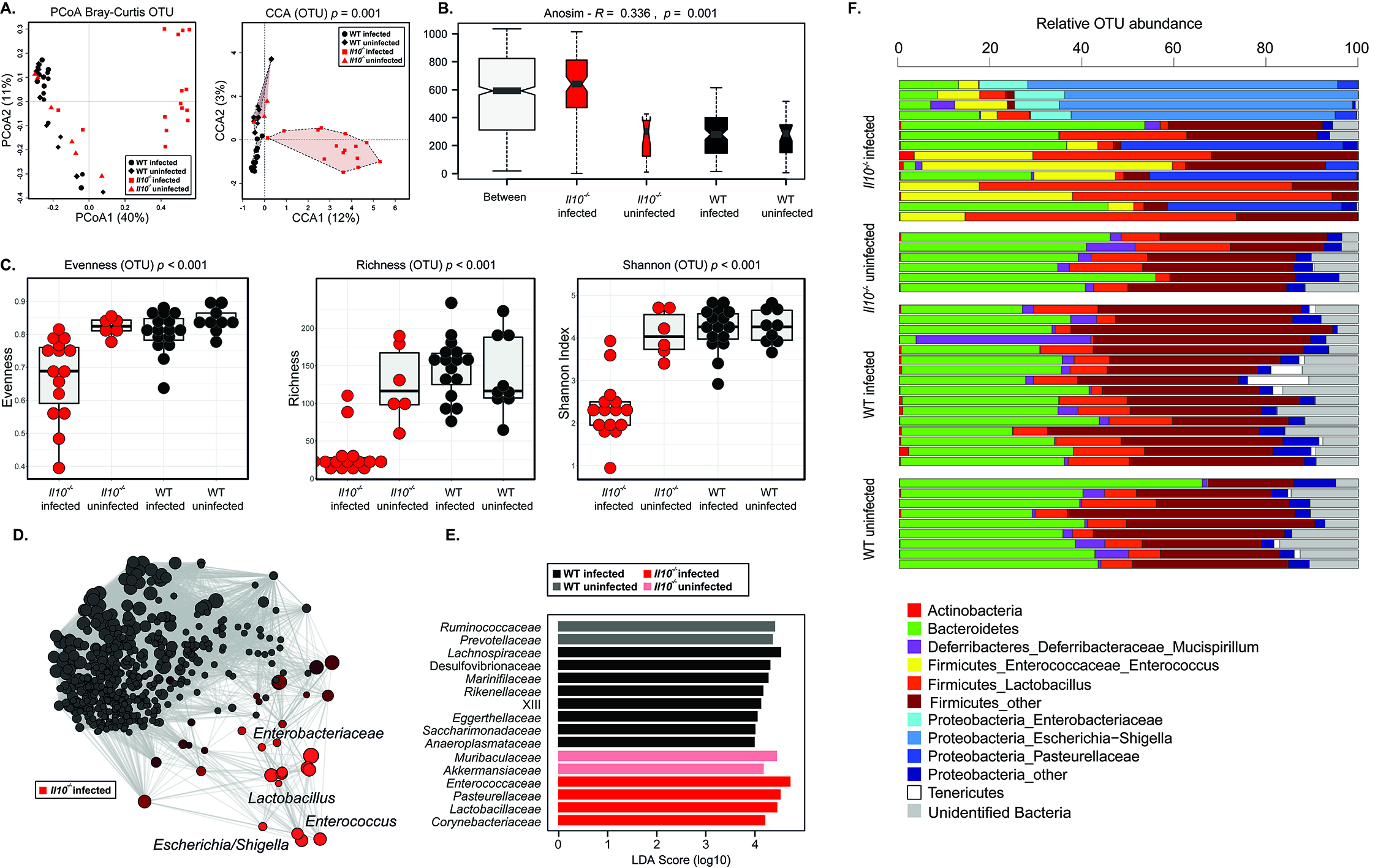

Supplement: S9 Fig — Caecal microbial community structure at the operational taxonomic unit (OTU) level of co-housed uninfected and T. muris-infected (high dose, 400 eggs) six-wk-old female and male littermate WT and Il10-/- mice at day of culling. (A) Principal Coordinates Analysis (PCoA) and Canonical Correspondence Analysis (CCA p = 0.001), the numbers in bracket indicate the percentage variance explained by that component, (B) beta-diversity index (ANOSIM R = 0.336 and p = 0.001), (C) alpha-diversity indexes (Shannon diversity, richness and evenness; ANOVA p<0.001, p<0.001, p<0.001, respectively), (D) network analysis, (E) Linear Discriminant Analysis Effect Size (LEfSe) analysis and (F) bar plots representing proportional abundance of individual OTUs in caecal microbial community structures. Data from two independent replicas. WT uninfected n = 9. WT infected n = 16. Il10-/- uninfected n = 6. Il10-/- infected n = 15. (TIF) [file ppat.1007265.s009.tif]

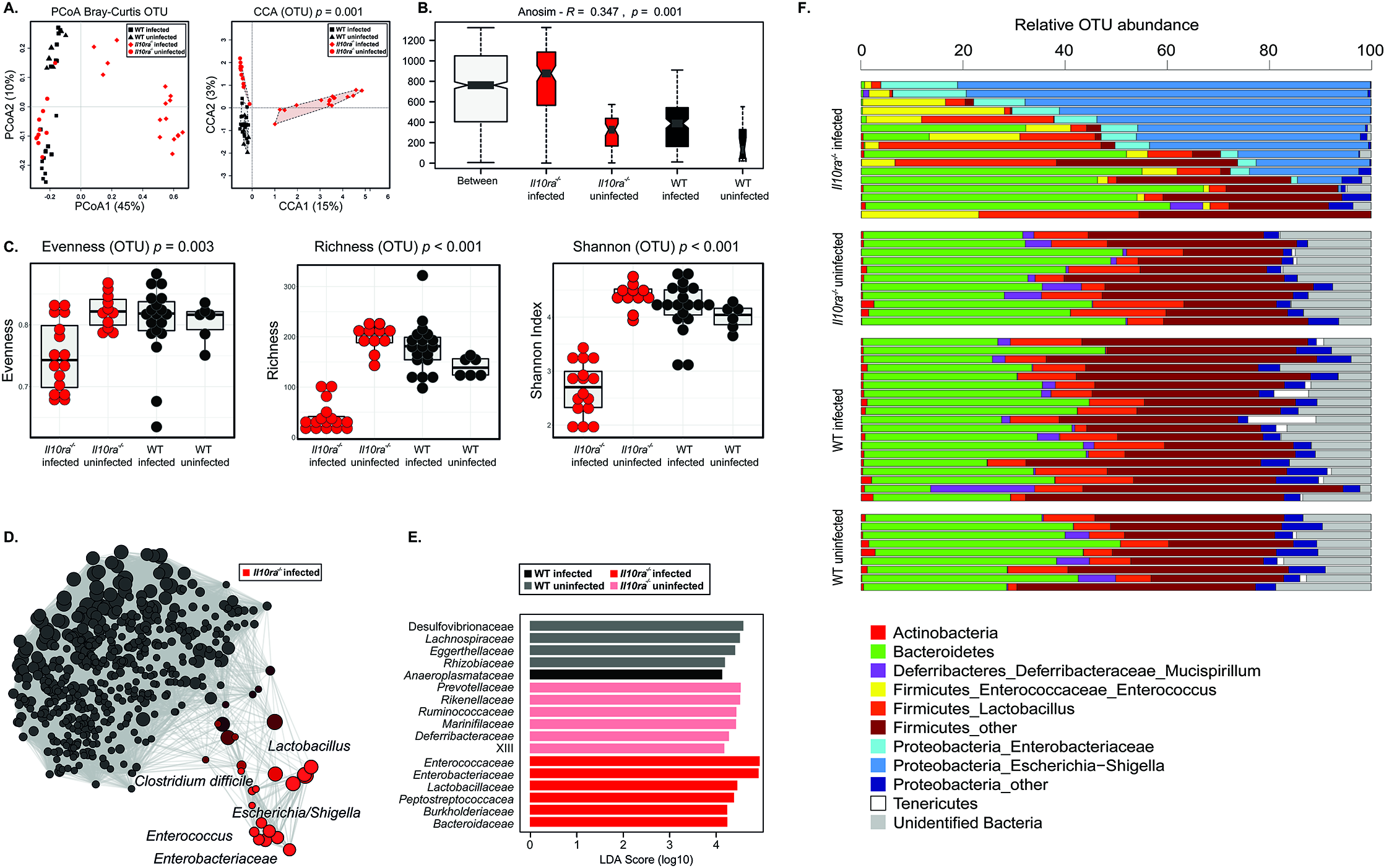

Supplement: S10 Fig — Caecal microbial community structure at the operational taxonomic unit (OTU) level of co-housed uninfected and T. muris-infected (high dose, 400 eggs) six-wk-old female and male littermate WT and Il10ra-/- mice at day of culling. (A) Principal Coordinates Analysis (PCoA) and Canonical Correspondence Analysis (CCA p = 0.001), the numbers in bracket indicate the percentage variance explained by that component, (B) beta-diversity index (ANOSIM R = 0.347 and p = 0.001), (C) alpha-diversity indexes (Shannon diversity, richness and evenness; ANOVA p = 0.003, p<0.001, p<0.001, respectively), (D) network analysis, (E) Linear Discriminant Analysis Effect Size (LEfSe) analysis and (F) bar plots representing proportional abundance of individual OTUs in caecal microbial community structures. Data from two independent replicas. WT uninfected n = 6. WT infected n = 19. Il10ra-/- uninfected n = 11. Il10ra-/- infected n = 16. (TIF) [file ppat.1007265.s010.tif]

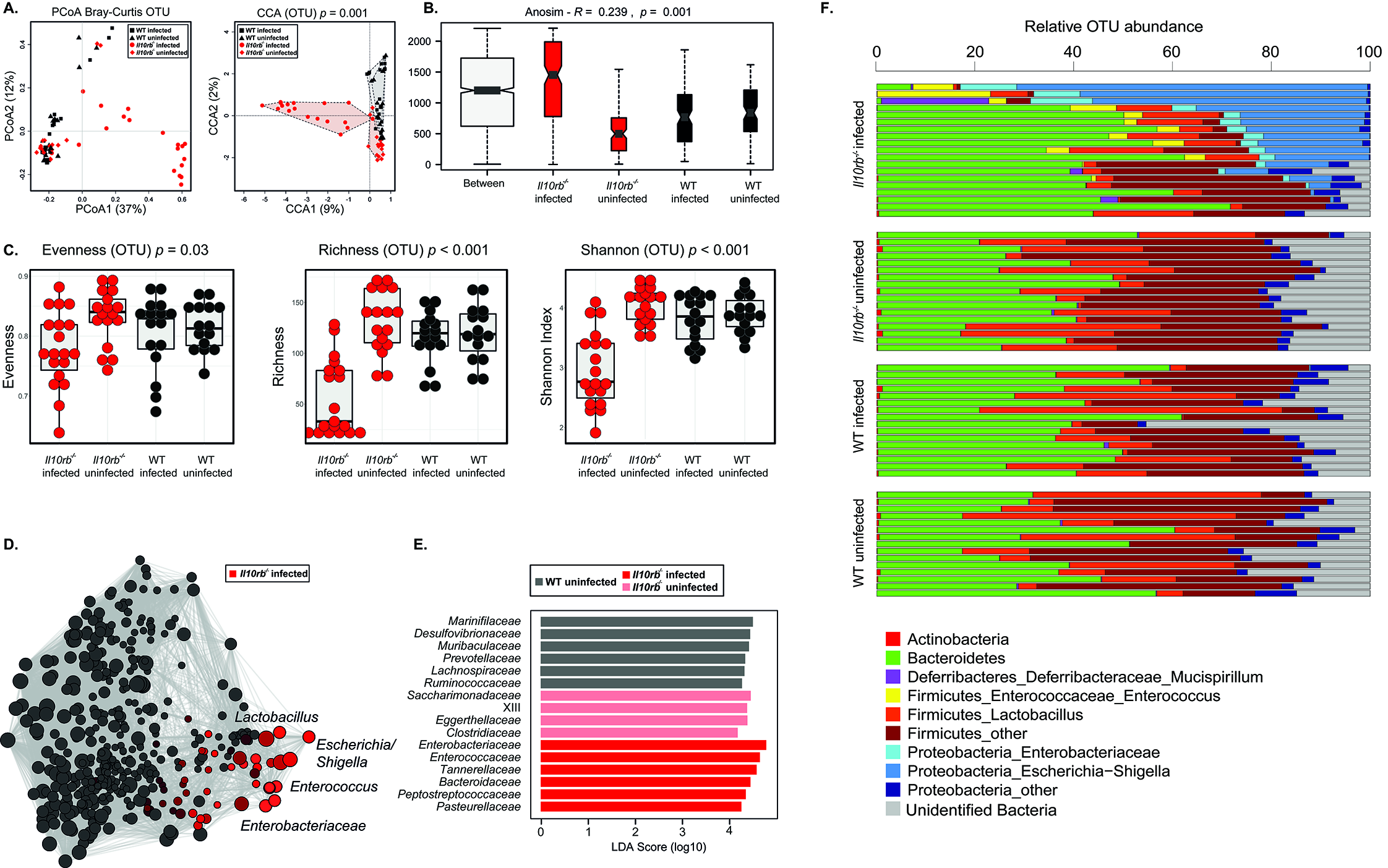

Supplement: S11 Fig — Caecal microbial community structure at the operational taxonomic unit (OTU) level of co-housed uninfected and T. muris-infected (high dose, 400 eggs) six-wk-old female and male littermate WT and Il10rb-/- mice at day of culling. (A) Principal Coordinates Analysis (PCoA) and Canonical Correspondence Analysis (CCA p = 0.001), the numbers in bracket indicate the percentage variance explained by that component, (B) beta-diversity index (ANOSIM R = 0.239 and p = 0.001), (C) alpha-diversity indexes (Shannon diversity, richness and evenness; ANOVA p = 0.03, p<0.001, p<0.001, respectively), (D) network analysis, (E) Linear Discriminant Analysis Effect Size (LEfSe) analysis and (F) bar plots representing proportional abundance of individual OTUs in caecal microbial community structures. Data from two independent replicas. WT uninfected n = 15. WT infected n = 16. Il10rb-/- uninfected n = 17. Il10rb-/- infected n = 19. (TIF) [file ppat.1007265.s011.tif]

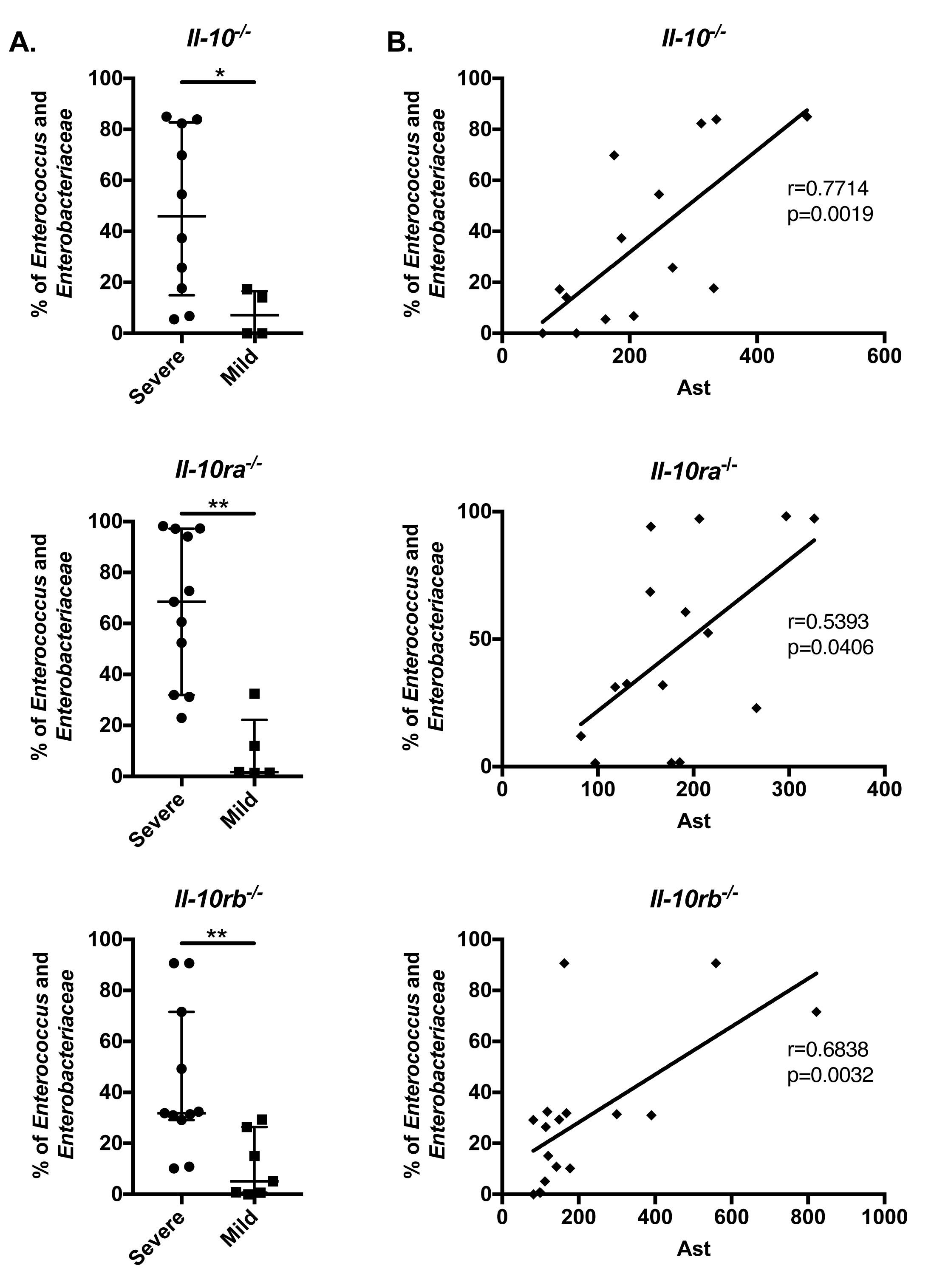

Supplement: S12 Fig — (A) The degree of colonization by Enterococcus and Enterobacteriaceae (percentage of abundance from total microbiota) among IL-10 signalling-deficient mice showing a severe or mild phenotype upon whipworm infection was compared. A severe phenotype is characterised by presence of granulomas, necrosis and foamy macrophages in the liver, weight loss and poor survival. A mild phenotype involves minor liver infiltration, no weight loss and extended survival. For percentage of Enterococcus and Enterobacteriaceae (including Escherichia-Shigella), median and interquartile range are shown. Mann Whitney U Test, **p<0.005, *p = 0.05. (B) Correlation analysis of the degree of colonization of the pathobionts and the plasma levels of the liver enzyme aspartate aminotransferase (Ast) was performed using a two-tailed Spearman correlation test with a 95% confidence interval. r, Spearman's rank correlation coefficient. Data from two independent replicas. n = 4–11 each group. (TIF) [file ppat.1007265.s012.tif]

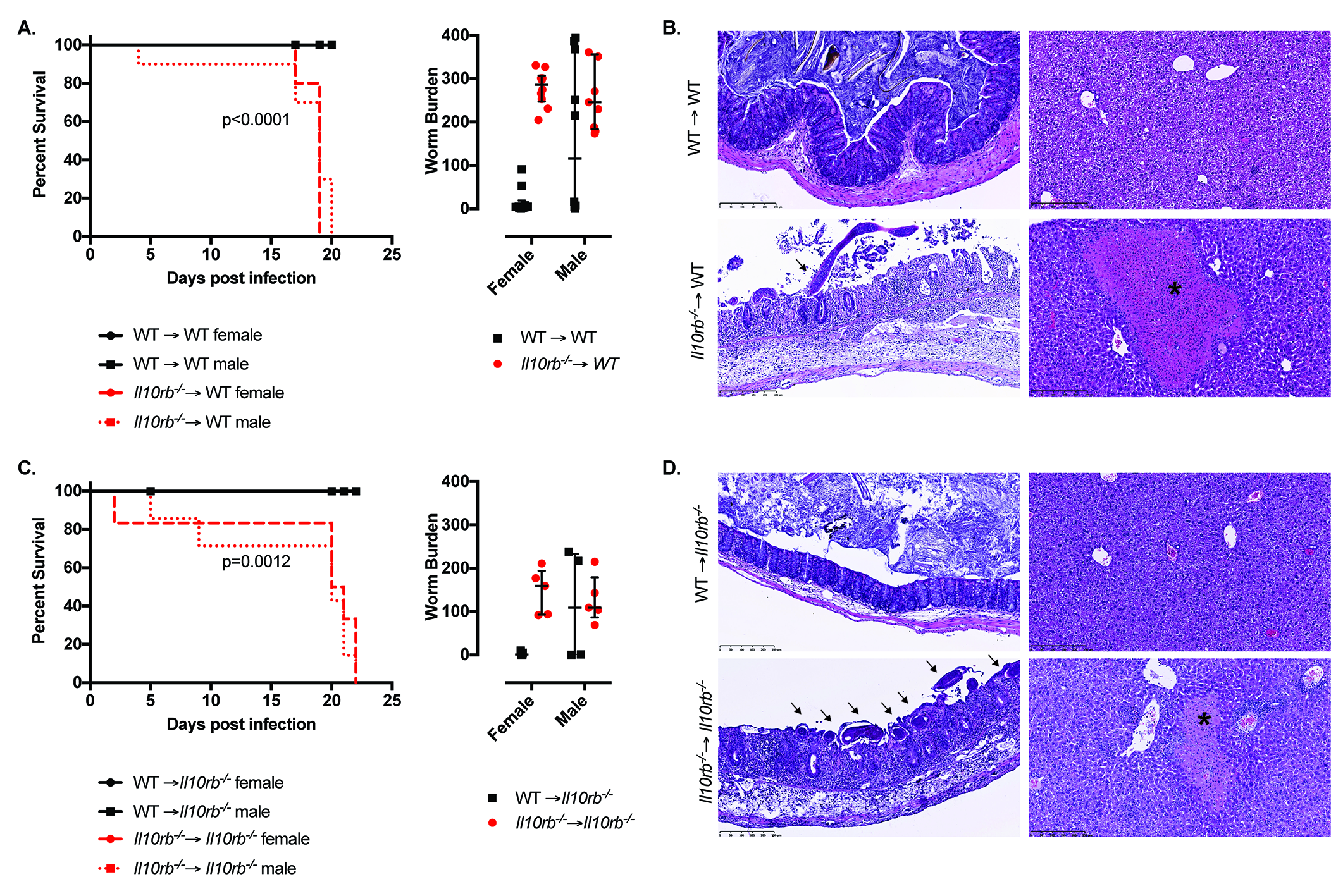

Supplement: S13 Fig — Survival curves, worm burdens and representative H&E histological images of T. muris-infected (high dose, 400 eggs) ten-wk-old female and male irradiated (A, B) WT and (C, D) Il10rb-/- mice reconstituted with the bone marrow of WT (black) or Il10rb-/- (red) mice. (A) Data from two independent replicas. WT → WT female n = 10. WT → WT male n = 10. Il10rb-/- → WT female n = 10. Il10rb-/- → WT male n = 10. For worm burdens, median and interquartile range are shown. Log-rank Mantel-Cox test for survival curves. (B) Data from two independent replicas. WT → Il10rb-/- female n = 7. WT → Il10rb-/- male n = 5. Il10rb-/- → Il10rb-/- female n = 5. Il10rb-/- → Il10rb-/- male n = 6. For worm burdens, median and interquartile range are shown. Log-rank Mantel-Cox test for survival curves. (B and D) T. muris worms infecting the mucosa are indicating with arrows and granulomatous lesions in the livers are indicated by asterisks. Scale bar, 250μm. (TIF) [file ppat.1007265.s013.tif]

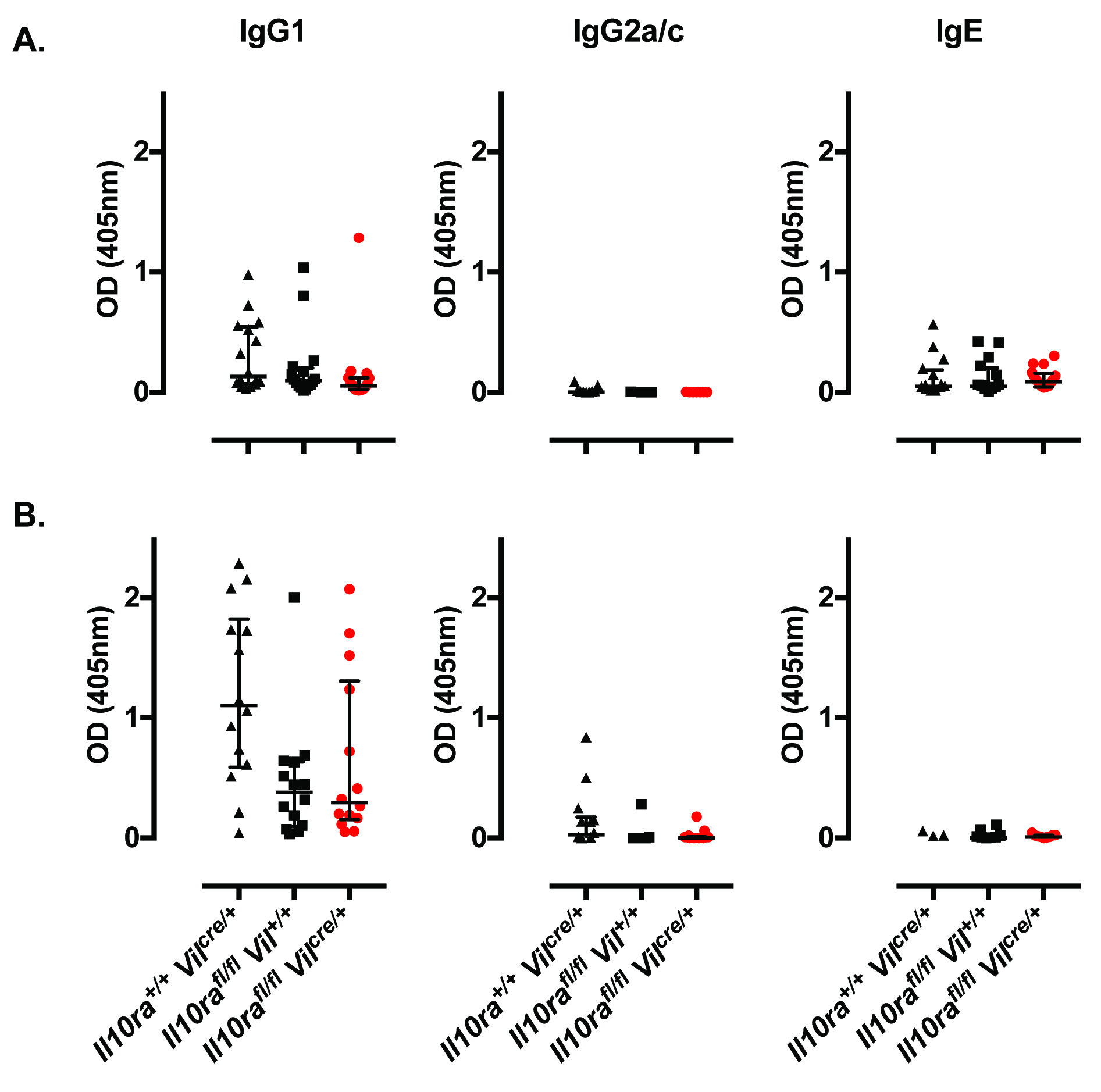

Supplement: S14 Fig — Antibody (IgG1, IgG2a/c and IgE) titers of T. muris-infected (high dose, 400 eggs) six-wk-old female and male littermates Il10ra+/+ Vilcre/+, Il10rafl/fl Vil+/+ and Il10rafl/fl Vilcre/+ mice after (A) 20 days (n = 16 mice for each group) and (B) 32 days of infection (n = 14 mice for each group). Data from two independent replicas. Median and interquartile range are shown. (TIF) [file ppat.1007265.s014.tif]
