# Supplementary material for: Exclusive dependence of IL-10Rα signalling on intestinal microbiota homeostasis and control of whipworm infection
Source: PLoS Pathog. 2019 Jan 14;15(1):e1007265. doi: 10.1371/journal.ppat.1007265 (PMC6347331; doi:10.1371/journal.ppat.1007265)
Supplement: S1 File — Extended information on housing and husbandry of mice and microbiota analysis is provided in this file. (DOCX) [file ppat.1007265.s015.docx]

# Supplemental Experimental Procedures

## Housing and husbandry of mice

Mice were maintained in a specific pathogen free unit on a 12hr light: 12hr dark cycle with lights off at 7:30pm and no twilight period.  The ambient temperature was 21 ± 2^o^C and the humidity was 55 ± 10%. Mice were housed for phenotyping using a stocking density of 3-5 mice per cage (overall dimensions of caging: (L x W x H) 365 x 207 x 140mm, floor area 530cm^2^) in individually ventilated caging (Techniplast Seal Safe1284L) receiving 60 air changes per hour. In addition to Aspen bedding substrate, standard environmental enrichment of two nestlets, a cardboard Fun Tunnel and three wooden chew blocks were provided.

## Microbiota Analysis

Raw paired-end Illumina reads were trimmed for 16S rRNA gene primer sequences using Cutadapt (<https://cutadapt.readthedocs.org/en/stable/>) and sequence data were processed using the Quantitative Insights Into Microbial Ecology 2 (QIIME2-2018.4; https://qiime2.org) software suite [1]. Successfully joined sequences were quality filtered, dereplicated, chimeras identified, and paired-end reads merged in QIIME2 using DADA2 [2]. Sequences were clustered into Operational Taxonomic Units (OTUs) on the basis of similarity to known bacterial sequences available in the SILVA database (<https://www.arb-silva.de/download/archive/qiime>; Silva_132); sequences that could not be matched to references in the SILVA database were clustered *de novo* based on pair-wise sequence identity (99% sequence similarity cut-off). The first selected cluster seed was considered as the representative sequence of each OTU. The OTU table with the assigned taxonomy was exported from QIIME2 alongside a weighted unifrac distance matrix. Singleton OTUs were removed prior to downstream analyses. Cumulative-sum scaling (CSS) was applied, followed by log2 transformation to account for the non-normal distribution of taxonomic counts data. Statistical analyses were executed using the Calypso software [3]; samples were clustered using Principal Coordinates Analysis (PCoA) and supervised Canonical Correspondence Analysis (CCA) including infection/mouse strain as explanatory variables and describing the percentage of associated variation explained. Differences in bacterial alpha diversity (Shannon diversity), richness and evenness between uninfected and *T. muris*-infected WT and IL-10, IL-10Rα and IL-10Rβ mutant mice, were automatically rarefied and evaluated using Analysis of Variance (ANOVA). Beta diversity was calculated using weighted UniFrac distances and differences in beta diversity were determined through Analysis of Similarity (ANOSIM); it compares the mean of ranked dissimilarities between groups to the mean of ranked dissimilarities within groups. An R value close to "1.0" suggests dissimilarity between groups while an R value close to "0" suggests an even distribution of high and low ranks within and between groups [4]. Correlation networks were constructed to identify clusters of co-occurring bacteria based on their association with the study groups (i.e., samples from uninfected and *T. muris*-infected WT and IL-10, IL-10Rα and IL-10Rβ mutant mice). Taxa and explanatory variables were represented as nodes, taxa abundance as node size, and edges represented positive associations, while nodes were coloured according to study group. Taxa abundances were associated with the different study groups using Pearson’s correlation, while nodes were coloured based on the strength of the association with each study group. Networks were generated by first computing associations between taxa using Spearman’s rho, followed by conversion of resulting pairwise correlations into dissimilarities. These were then used to ordinate nodes in a two-dimensional plot by PCoA. Therefore, correlating nodes were located in close proximity and anti-correlating nodes were placed at distant locations in the network. Differential abundance of microbial taxa between groups were assessed using the Linear discriminant analysis Effect Size (LEfSe) workflow [5] for caecal microbiota sequences, while differential abundance of specific bacterial taxa were evaluated through Kruskal-Wallis Test for liver microbiota sequences. Bar plots describing the taxa identified were generated excluding those with <0.2% abundance and sorted based on genotype, infection status and abundance of *E. coli*.

1. Caporaso JG, Kuczynski J, Stombaugh J, Bittinger K, Bushman FD, Costello EK. QIIME allows analysis of high-throughput community sequencing data. Nature Meth. 2010;7.

2. Callahan BJ, McMurdie PJ, Rosen MJ, Han AW, Johnson AJ, Holmes SP. DADA2: High-resolution sample inference from Illumina amplicon data. Nat Methods. 2016;13(7):581-3.

3. Zakrzewski M, Proietti C, Ellis JJ, Hasan S, Brion MJ, Berger B, et al. Calypso: a user-friendly web-server for mining and visualizing microbiome-environment interactions. Bioinformatics. 2017;33(5):782-3.

4. Clarke KR. Non-parametric multivariate analyses of changes in community structure. Austral Ecology. 1993;18(1):117-43.

5. Segata N, Izard J, Waldron L, Gevers D, Miropolsky L, Garrett WS.Metagenomic biomarker discovery and explanation. Genome Biol. 2011;12.
